# Early Experience with Acuson AcuNav 4D-ICE to Guide Transcatheter Tricuspid Edge-to-Edge Repair: 4D Intracardiac Echocardiography Compared to Transesophageal Echocardiography

**DOI:** 10.3390/jcdd12050165

**Published:** 2025-04-23

**Authors:** Matteo Biroli, Fabio Fazzari, Francesco Cannata, Vincenzo De Peppo, Cristina Ferrari, Carlo Maria Giacari, Marco Gennari, Paolo Olivares, Manuela Muratori, Mauro Pepi, Gianluca Pontone, Federico De Marco

**Affiliations:** 1 Department of Biomedical, Surgical and Dental Sciences, Università degli Studi di Milano, 20122 Milan, Italy; birolimatteo@gmail.com (M.B.); vincenzo.depeppo@unimi.it (V.D.P.); gianluca.pontone@cardiologicomonzino.it (G.P.); 2 Department of Peri-Operative Cardiology and Cardiovascular Imaging, Centro Cardiologico Monzino IRCCS, 20138 Milan, Italy; francesco.cannata@cardiologicomonzino.it (F.C.); manuela.muratori@cardiologicomonzino.it (M.M.); mauro.pepi@cardiologicomonzino.it (M.P.); 3 Department of Interventional, Valvular and Structural Heart Cardiology, Centro Cardiologico Monzino IRCCS, 20138 Milan, Italy; cristina.ferrari@cardiologicomonzino.it (C.F.); carlo.giacari@gmail.com (C.M.G.); marco.gennari@cardiologicomonzino.it (M.G.); paolo.olivares@cardiologicomonzino.it (P.O.); federico.demarco@gmail.com (F.D.M.)

**Keywords:** intracardiac echocardiography, tricuspid regurgitation, tricuspid edge-to-edge repair, 3D echocardiography, structural heart diseases

## Abstract

Tricuspid regurgitation is a common valvular disease associated with high morbidity and mortality if left untreated. While surgery has been the standard intervention, transcatheter tricuspid edge-to-edge repair (T-TEER) has emerged as an alternative for high-risk surgical candidates. Transesophageal echocardiography (TEE) is the gold-standard imaging modality for guiding T-TEER due to its high spatial and temporal resolution. However, it requires general anesthesia and esophageal intubation, limiting its use in certain patients. Additionally, TEE image quality may be compromised by anterior structure shadowing, which is common in T-TEER. The development of 4D intracardiac echocardiography (ICE) offers real-time, three-dimensional imaging, potentially overcoming these limitations. This study compared TEE and Acuson AcuNav 4D-ICE in guiding T-TEER in ten high-risk patients across eight crucial procedural steps. ICE showed optimal feasibility in key procedural steps, including valve steering and leaflet grasping, due to its proximity to target structures, minimizing shadowing artifacts. Both modalities performed equally in lesion identification and residual regurgitation assessment and achieved non-statistically different results in most quantitative measurements. This study supports the integration of 4D-ICE into T-TEER procedures, particularly for patients unsuited for TEE or with complex TEE windows. Its real-time imaging, reduced invasiveness, and feasibility in critical steps highlight its potential as a viable alternative or complement to TEE. Further multicenter studies are needed to validate its role, optimize protocols, and evaluate long-term outcomes in 4D-ICE-guided T-TEER.

## 1. Introduction

Tricuspid regurgitation (TR) is a prevalent valvular heart disease that can lead to severe right heart failure and significant morbidity and mortality if left untreated [1].

While surgery has traditionally been the unique interventional treatment for severe TR, the development of percutaneous treatment options, such as tricuspid transcatheter edge-to-edge repair (T-TEER), has provided a valuable alternative for a wide cohort of patients deemed at high risk for surgical interventions. These transcatheter procedures necessitate precise and reliable imaging to ensure proper device placement and procedural success [1].

Transesophageal echocardiography (TEE) is the key imaging technique for the intraprocedural guidance of transcatheter tricuspid valve interventions due to its high spatial and temporal resolution [1,2,3].

However, the requirement for general anesthesia and esophageal intubation inherent in TEE renders it unsuitable for certain patient populations, particularly those with esophageal disorders or those at elevated risk for anesthesia-related complications [4]. In addition, a clear visualization of tricuspid valve anatomy and details may be challenging due to the anterior location of the valve and, in some cases, the presence of aortic or mitral prostheses [5].

Hence, there is a need for adjunctive imaging modalities to enhance visualization and facilitate guidance of transcatheter procedures. Although two-dimensional intracardiac echocardiography (ICE) imaging has been proven to be successful in T-TEER procedures, more advanced intracardiac probes have been emerging as a crucial technological innovation in the guidance of structural heart interventions [5].

ICE employs catheters equipped with ultrasound probes that are introduced into the heart’s chambers, delivering unique and detailed perspectives from within the cardiac anatomy [5,6]. This modality can facilitate precise device deployment during interventions and can be performed under conscious sedation, thereby potentially mitigating procedural risks [5,7]. The advent of 4D-ICE technology, which provides real-time three-dimensional imaging, constitutes a significant enhancement, offering dynamic views that improve visualization of the tricuspid valve and adjacent structures during T-TEER procedures [5,7].

This study aims to conduct a comparative analysis of TEE and Acuson AcuNav 4D-ICE (Siemens Healthineers, Germany) performance in the context of T-TEER. As of 2024, at the time of this study, this device was the only 4D-ICE system approved for commercial use in the European market.

By assessing the imaging quality and procedural guidance capabilities, this manuscript endeavors to ascertain potential strengths, weaknesses, and common features of each modality for guiding T-TEER procedures.

## 2. Materials and Methods

A cohort of ten consecutive patients undergoing T-TEER was prospectively enrolled for this study. The selection criteria included patients with symptomatic severe tricuspid regurgitation deemed at high risk for conventional surgery. Ethical approval was obtained from the institutional review board, and informed consent was secured from all participants.

Each patient simultaneously received both TEE and 4D-ICE during the T-TEER procedure. This dual-imaging approach was intended to allow for a comprehensive comparison of the imaging modalities throughout the intervention.

The 4D-ICE images were acquired with Acuson SC2000 PRIME equipped with Acuson AcuNav Volume ICE catheter (Siemens Healthineers, Forchheim, Germany); all TEEs were acquired using Philips Epiq 7 with ×8 probe (Philips Ultrasound; Botherl, WA, USA). Images were analyzed using post-processing software (TomTec-Arena version 2.30; Unterschleibheim, Germany).

### 2.1. Insights into Acuson AcuNav 4D—ICE

**Figure 1:**
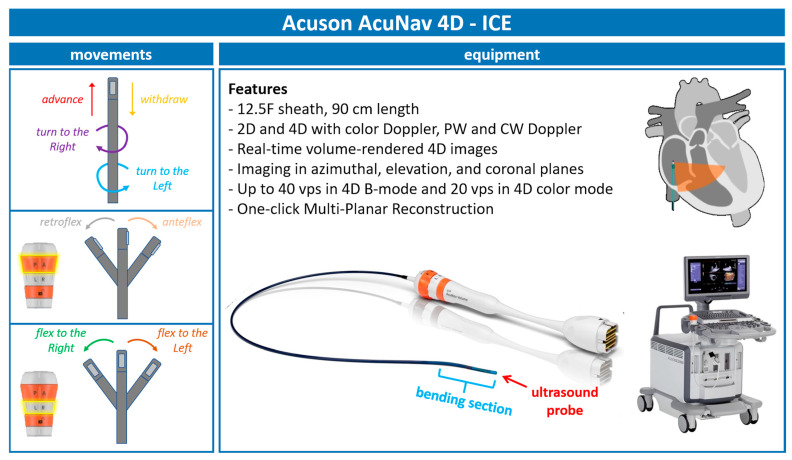
**Acuson—AcuNav 4D-ICE characteristics**. Acuson AcuNav 4D-ICE catheter system, with highlights on its design, technical details, and movements. Abbreviations: 4D-ICE: 4D intracardiac echocardiography.

With continuous advancements in ICE technology, we are now utilizing the 5th generation of AcuNav ICE probes (Figure 1). The latest iteration, the Acuson AcuNav Volume ICE catheter, offers both 2D and 4D imaging capabilities, including color Doppler flow, pulsed wave (PW), and continuous wave (CW) spectral Doppler. This probe features a 12.5F sheath and measures 90 cm in length.

Additionally, this probe provides the following:

- Real-time volume-rendered 4D images;
- Imaging in azimuthal, elevation, and coronal planes;
- Up to 40 volumes per second (vps) in 4D B-mode and 20 vps in 4D color mode;
- One-click multiplanar reconstruction (MPR).

### 2.2. 4D-ICE Movements

To effectively use the ICE catheter during procedures, it is important to follow specific movements and positioning steps, ensuring clear visualization and precise guidance.

The procedure begins with the insertion of the 4D-ICE catheter through the femoral vein using an introducer sheath. Once advanced into the right atrium, the catheter is positioned to acquire the home view and then rotated clockwise until the aorta–right ventricular outflow tract view is obtained. The catheter is then flexed toward the tricuspid valve (TV) to achieve an anterior–posterior orientation along the probe’s long axis, offering a comprehensive view of the valve structure.

From this position, 3D MPR is used to sweep across the TV. This allows for the visualization of the valve leaflets, the identification of regurgitation jets, and the assessment of leaflet lengths and coaptation gaps. For optimal imaging, adjustments to the catheter’s flexion or tip rotation are necessary, aligning the probe to maximize the resolution and ensure accurate anatomical views.

During leaflet grasping, the ICE probe plays a crucial role in confirming that the leaflets lie flat on the device’s arms and that their tips are centrally positioned within the device. To verify the alignment and orientation of the device, fluoroscopic imaging is combined with ICE, ensuring that the clip arms are visible in both open and closed positions without parallax distortion.

**Figure 2:**
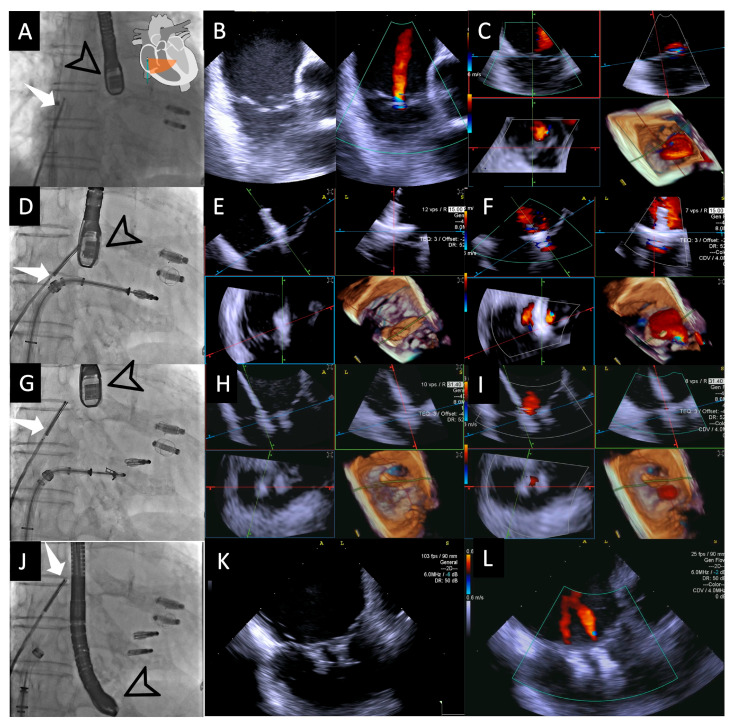
**Fluoroscopic and intracardiac views during T-TEER**. Fluoroscopic image (**A**) showing both the 4D-ICE probe (white arrow) and TEE probe (black arrow); the two previously implanted M-TEER devices are visible in this patient. In this case, the ICE probe is positioned directly facing the tricuspid valve, allowing for the first evaluation of the valve through 2D images without and with color Doppler (**B**), followed by MPR (**C**), which will serve as our “Home View” throughout the entire procedure. Fluoroscopic image (**D**) showing the implantation of the first T-TEER device, which is also visualized in MPR as the leaflets are grasped, with clip stability assessed without color (**E**) and residual jets evaluated with color Doppler (**F**). Fluoroscopic image (**G**) showing the implantation of the second edge-to-edge device monitored again through MPR without color (**H**) and with color Doppler (**I**). Fluoroscopic image (**J**) during the final evaluation of results, with a 2D image without color confirming good device stability (**K**) and a 2D image with color Doppler demonstrating mild residual regurgitant jets (**L**). Abbreviations: 4D-ICE: 4D intracardiac echocardiography. MPR: multiplanar reconstruction. M-TEER: mitral transcatheter edge-to-edge repair; T-TEER: tricuspid transcatheter edge-to-edge repair.

Once the device is deployed, 4D-ICE is used for a final assessment. This includes confirming proper leaflet insertion, evaluating the bridging of the leaflets, and measuring residual gradients or orifice areas using MPR. Additionally, the reduction in tricuspid regurgitation is confirmed through detailed imaging. All these steps are highlighted in Figure 2.

### 2.3. Procedural Steps

The T-TEER procedure was systematically divided into eight steps, each critical to the success of the intervention. At each procedural step, TEE and 4D-ICE images were acquired and analyzed independently after the procedure by two experienced echocardiographers who were blinded to each other’s assessments. The quality of images, the clarity of anatomical details, and the ability in guiding each procedural step were evaluated for each individual imaging modality.

The identified steps were as follows:

- Step number 1: assessment of tricuspid valve anatomy.
- Step number 2: identification of the target lesion.
- Step number 3: steering and valve approach.
- Step number 4: ensuring perpendicularity and correct trajectory.
- Step number 5: clocking.
- Step number 6: grasping.
- Step number 7: leaflet insertion.
- Step number 8: evaluation of residual regurgitant jets.

If additional devices were necessary, the preceding steps were repeated to achieve optimal outcomes.

**Figure 3:**
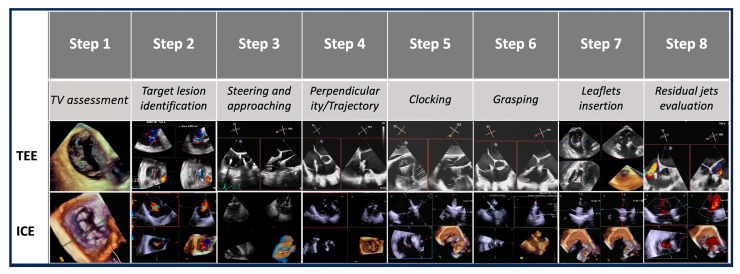
**Procedural steps**. This figure illustrates the procedural workflow for tricuspid valve intervention using echocardiographic guidance across the eight procedural steps. Abbreviations:. TV: tricuspid valve. ICE: intracardiac echocardiography. TEE: transesophageal echocardiography.

Evaluated steps are summarized in Figure 3.

### 2.4. Endpoints

#### 2.4.1. Feasibility/Image Quality

The feasibility of using a single imaging modality for intraprocedural guidance was evaluated based on its capability to independently provide high-quality images sufficient for guiding the procedure. This assessment was conducted after the procedure in three grades by two echocardiographers who were blinded to the procedure outcomes. The grading was as follows:

- Excellent (+++): The image quality was deemed sufficient to complete the procedural step independently without the need for additional images from another modality.
- Good (++): The image quality was adequate to complete the procedural step; however, verification with an alternative imaging modality could potentially enhance the outcome.
- Poor (+): The image quality was insufficient for completing the procedural step without difficulties, necessitating the use of another imaging modality.

Imaging modalities performance in cases in which more than one edge-to-edge device was necessary was re-evaluated focusing on step 6 (grasping) and step 7 (leaflet insertion).

#### 2.4.2. Leaflet and Annulus Assessments

**Figure 4:**
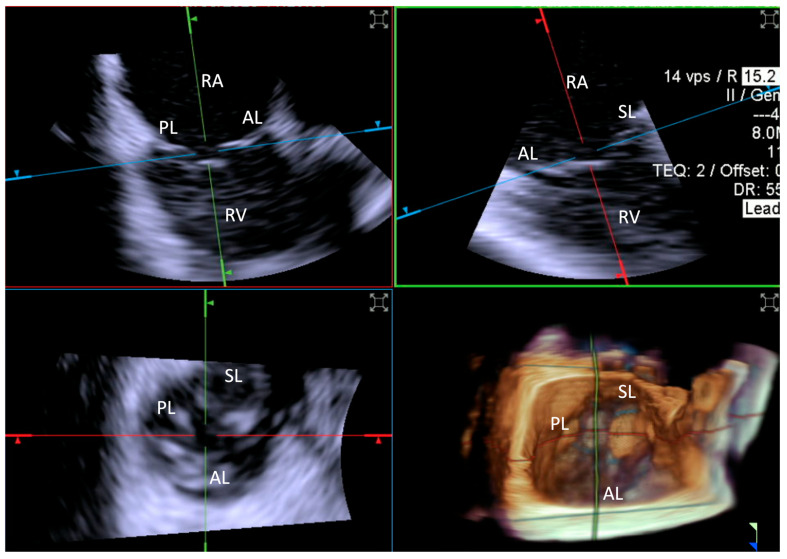
**Home view**. Home View refers to the primary imaging reference used with 4D-ICE throughout the T-TEER procedure, providing continuous monitoring of the tricuspid valve and device positioning. This view is generated through MPR, ensuring optimal visualization of anatomical structures and procedural steps. Initially, the evaluation is performed using 2D imaging without and with color Doppler, allowing for the assessment of leaflet motion and blood flow. The Home View remains the central imaging perspective during leaflet grasping, clip deployment, and post-implantation stability assessment, ensuring precise procedural guidance and real-time decision-making. Abbreviations: 4D-ICE: four-dimensional intracardiac echocardiography. AL: anterior leaflet. MPR: multiplanar reconstruction. PL: posterior leaflet. SL: septal leaflet. RA: right atrium. RV: Right Ventricle. T-TEER: tricuspid transcatheter edge-to-edge repair.

Leaflet length was measured by TEE in the mid-esophageal position. This involved the acquisition of 3D zoom–volume images, complemented by MPR at the level of each leaflet, enabling detailed visualization and measurement from the base to the free edge. For ICE evaluations, comparable 3D zoom–volume images were obtained from the standard “home view” (Figure 4). This method was employed to ensure consistency across imaging modalities, facilitating accurate comparisons. Measurements encompassed the anterior, septal, and posterior leaflets.

Furthermore, the annular perimeter, as well as the septal–lateral and anteroposterior diameters, were quantified using 3D volumes generated by both TEE and ICE.

#### 2.4.3. Quantification of Tricuspid Regurgitation Before and After Procedure

Quantification of TR was achieved with 2D vena contracta (VC) and number of residual jets pre- and post-procedure. TR was divided into six grades: mild, mild to moderate, moderate to severe, severe, massive, and torrential. Final trans-tricuspid gradients were compared as well.

### 2.5. Statistical Analysis

Categorical variables are reported as numbers (percentage), while continuous variables are reported as mean (±SD). To compare measurements of continuous data obtained using 4D-ICE and TEE, we utilized the Wilcoxon signed-rank test for paired data. In cases where the data were incomplete or unpaired, the Mann–Whitney U test was employed as a non-parametric alternative to assess differences between the two groups. Two-sided *p* values < 0.05 were considered statistically significant. Statistical analyses were performed using IBM Corp. Released 2023. IBM SPSS Statistics for Macintosh, Version 29.0.2.0 Armonk, NY, USA: IBM Corp.

## 3. Results

### 3.1. Baseline Clinical and Echocardiographic Characteristics

Ten patients were included in our study. Females were 50%, while the mean age was 77 ± 9.9 years.

The mean Triscore was 19%. Previous mitral valve replacement was performed in three out of ten patients, two had previously undergone mitral TEER and one patient had a history of aortic valvuloplasty coupled with ascending aorta replacement.

The baseline clinical characteristics of the patients are summarized in Table 1.

Tricuspid regurgitation was severe in 9/10 of patients and massive in 1 out of 10. Regarding the mechanism of tricuspid regurgitation (TR), 2/10 patients had primary TR, 8/10 had secondary TR, and none had CIED-related TR. Atrial functional TR was present in 100% of patients with secondary TR. Right atrium areas showed significant variability, with a mean of 36.1 cm^2^.

Echocardiographic parameters are summarized in Table 2.

### 3.2. Procedural Details

All procedures were performed via the right femoral approach, which was closed with a suture-based vascular closure device in all cases (Prostyle–Abbott Vascular). ICE-related access was the right femoral vein in 100% of cases. The devices used in the procedure were equally distributed between Pascal (Edwards LifeScience) (50%) and TriClip (Abbott Vascular) (50%). The distribution of the number of clips required per procedure varied, with the majority of patients needing two clips (70%). The mean procedural time was noted to be 91.8 min with a standard deviation of 13.9 min, indicating a relatively consistent procedure duration across cases. Post-procedure, the majority of patients experienced mild regurgitation (80%), with a smaller subset experiencing mild–moderate regurgitation (20%). There were no procedural complications, vascular complications, or device failures. Procedural details are reported in Table 3.

### 3.3. Main Results—TEE vs. 4D-ICE Comparison

#### 3.3.1. Step-by-Step Evaluation

While both modalities demonstrated utility, their performance varied significantly across the procedural steps:

- Excellent (+++) Ratings: ICE achieved 76 Excellent ratings out of 80 steps, compared to 56 Excellent ratings for TEE.
- Good (++) Ratings: ICE and TEE achieved “Good” ratings in 4 steps and 10 steps, respectively, reflecting situations where imaging was adequate but could have been improved with an alternative modality.
- Poor (+) Ratings: ICE did not receive any “Poor” ratings, whereas TEE required complementary imaging in 14 instances.

*Step 1: Assessment of Tricuspid Valve Anatomy*

- ICE: 8 Excellent (+++), 2 Good (++), and 0 Poor (+)
- TEE: 6 Excellent (+++), 4 Good (++), and 0 Poor (+)

Both modalities performed well in this step, with ICE achieving slightly more “Excellent” ratings. TEE was adequate in all cases, but “Good” ratings were more frequent compared to ICE.

**Figure 5:**
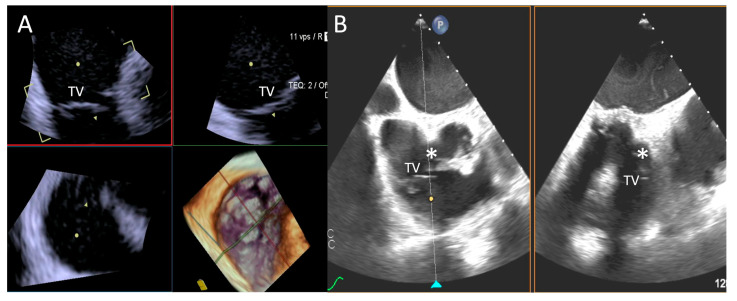
**Step 1: insights from Case 4.** The figure shows a comparative analysis of the tricuspid valve morphology evaluation during Step 1. In this case, the 4D-ICE (**A**) system shows better image quality in assessing TV morphology than TEE (**B**) due to anterior structures shadowing (*). Abbreviations: 4D-ICE: four-dimensional intracardiac echocardiography. TEE: transesophageal echocardiography. TV: tricuspid valve.

Reasons for sub-optimal ICE image were mainly related to a large annulus that could not completely be visualized by 4D-ICE, whereas TEE image quality was affected by shadowing artifacts or poor trans-gastric views. An example is provided in Figure 5.

*Step 2: Identification of the Target Lesion*

- ICE: 10 Excellent (+++), 0 Good (++), and 0 Poor (+)
- TEE: 10 Excellent (+++), 0 Good (++), and 0 Poor (+)

Both ICE and TEE achieved universally “Excellent” ratings in this step, demonstrating their reliability in identifying the target lesion.

*Step 3: Steering and Valve Approach*

- ICE: 10 Excellent (+++), 0 Good (++), and 0 Poor (+)
- TEE: 7 Excellent (+++), 0 Good (++), and 3 Poor (+)

**Figure 6:**
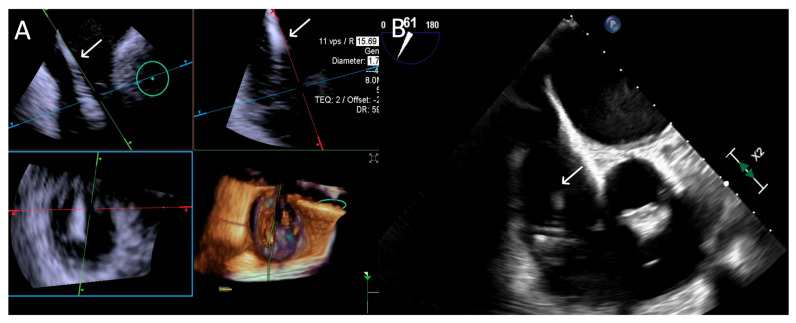
**Step 3: insights from Case 4.** The figure illustrates the comparative analysis of steering performance during Step 3. In this case, the superior performance of the 4D-ICE (**A**) system is shown, as compared to TEE (**B**). This highlights how the optimal use of the 4D-ICE system leads to enhanced steering catheter (white arrow) precision and control. In this particular scenario, the ICE system proved to be the most effective solution, offering notable advantages to guide the procedure. Green circle identifies the position of the aortic valve. Abbreviations: 4D-ICE: four-dimensional intracardiac echocardiography. TEE: transesophageal echocardiography.

ICE provided consistently excellent imaging, whereas TEE exhibited variability, with three cases rated as “Poor”, which was again related to shadowing of anterior structures, requiring complementary imaging for this critical step. An example is provided in Figure 6.

*Step 4: Ensuring Perpendicularity and Correct Trajectory*

- ICE: 10 Excellent (+++), 0 Good (++), and 0 Poor (+)
- TEE: 7 Excellent (+++), 0 Good (++), and 3 Poor (+)

**Figure 7:**
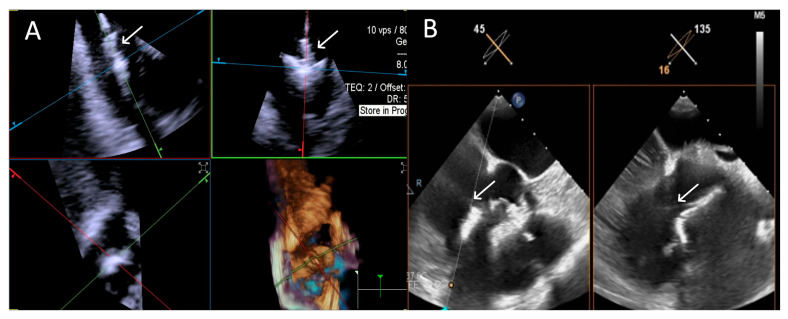
**Step 4: insights from Case 3.** The figure shows a comparative analysis of the trajectory evaluation of the delivery catheter (white arrow) during Step 4. In this case, the 4D-ICE (**A**) system offers better image quality, as TEE (**B**) was affected by shadowing artifacts from anterior structures. Abbreviations: 4D-ICE: four-dimensional intracardiac echocardiography. TEE: transesophageal echocardiography.

ICE demonstrated robust performance with 100% “Excellent” ratings. TEE showed similar challenges as in Step 3. An example is provided in Figure 7.

*Step 5: Clocking*

- ICE: 10 Excellent (+++), 0 Good (++), and 0 Poor (+)
- TEE: 9 Excellent (+++), 0 Good (++), and 1 Poor (+)

**Figure 8:**
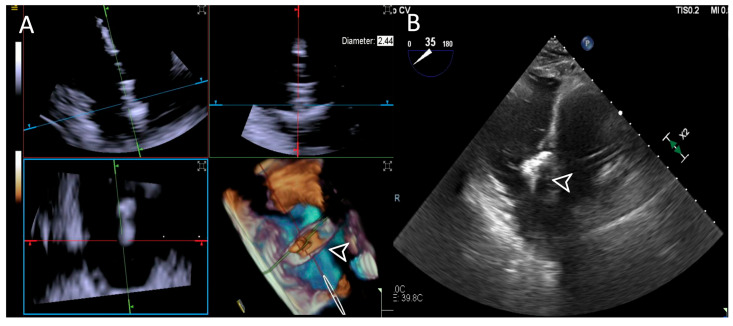
**Step 5: insights from Case 8.** The figure illustrates the comparative analysis of device (white arrowhead) clocking evaluation during Step 5. In this case, the 4D-ICE (**A**) system shows superior quality compared to the TEE (**B**) system due to poor trans-gastric view in TEE images which affected its performance. Abbreviations: 4D-ICE: four-dimensional intracardiac echocardiography. TEE: transesophageal echocardiography.

Both modalities performed well in this step, with ICE achieving “Excellent” ratings across all cases and TEE slightly underperforming with a “Poor” rating in one case due to an absent trans-gastric view. An example is provided in Figure 8.

*Step 6: Grasping*

- ICE: 9 Excellent (+++), 1 Good (++), and 0 Poor (+)
- TEE: 4 Excellent (+++), 3 Good (++), and 3 Poor (+)

**Figure 9:**
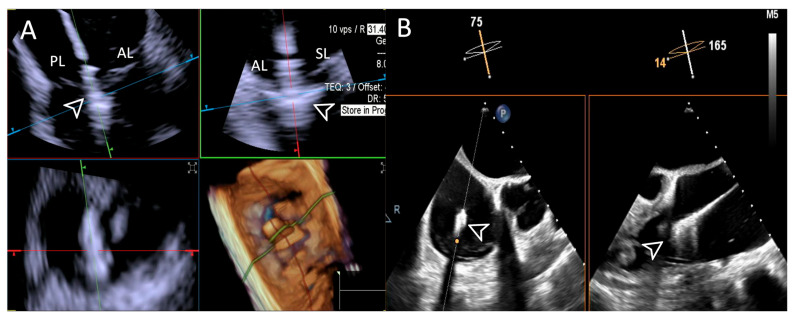
**Step 6: insights from Case 1.** The figure illustrates the comparative analysis of leaflet grasping (white arrowhead) evaluation during Step 6. In this case, the superior performance of the 4D-ICE (**A**) system is shown, as compared to TEE, (**B**) due to shadowing artifacts from anterior structures. Abbreviations: 4D-ICE: four-dimensional intracardiac echocardiography. AL: anterior leaflet. PL: posterior leaflet. SL: septal leaflet. TEE: transesophageal echocardiography.

ICE provided high-quality imaging with no “Poor” ratings. TEE demonstrated greater variability, with three cases rated as “Poor” and requiring complementary imaging. Shadowing was again regarded as the main issue as far as TEE was concerned. An example is provided in Figure 9.

*Step 7: Leaflet Insertion*

- ICE: 9 Excellent (+++), 1 Good (++), and 0 Poor (+)
- TEE: 3 Excellent (+++), 3 Good (++), and 4 Poor (+)

**Figure 10:**
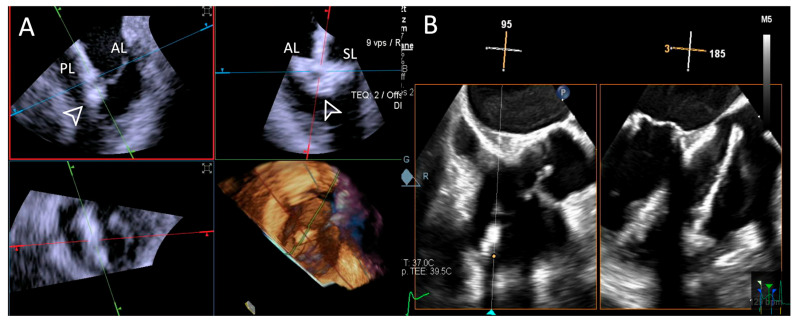
**Step 7: insights from Case 6.** The figure illustrates the comparative analysis of leaflet insertion (white arrowhead) evaluation during Step 7. In this case, the superior performance of the 4D-ICE (**A**) system is shown, as compared to TEE, (**B**) due to shadowing from anterior structures. Abbreviations: 4D-ICE: four-dimensional intracardiac echocardiography. AL: anterior leaflet. PL: posterior leaflet. SL: septal leaflet. TEE: transesophageal echocardiography.

**Figure 11:**
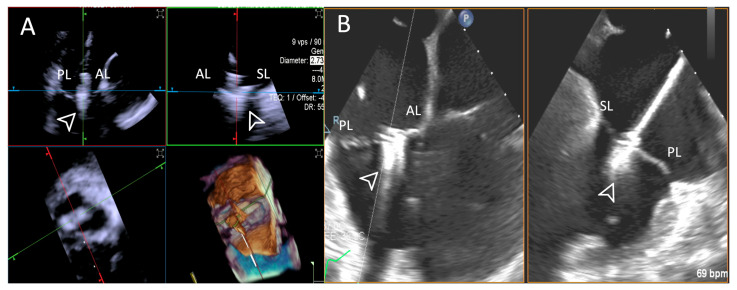
**Step 7: insights from Case 10**. The figure illustrates the comparative analysis of leaflet insertion (white arrowhead) evaluation during Step 7. In this case, the superior performance of the TEE (**B**) system is shown, as compared to 4D-ICE (**A**), due to TEE superior spatial and temporal resolution and large tricuspid valve annulus that extended beyond the field of view of the 4D-ICE imaging. Abbreviations: 4D-ICE: four-dimensional intracardiac echocardiography. AL: anterior leaflet. PL: posterior leaflet. SL: septal leaflet. TEE: transesophageal echocardiography.

Leaflet insertion was another step where ICE performed better than TEE, achieving more “Excellent” ratings. TEE struggled in this step due to the same issues as the previous step. This was particularly related to a clearer visualization of the septal leaflet by 4D-ICE. An example is provided in Figure 10 and Video S1. However, TEE had better performance in some cases, as highlighted in Figure 11.

*Step 8: Evaluation of Residual Regurgitant Jets*

- ICE: 10 Excellent (+++), 0 Good (++), and 0 Poor (+)
- TEE: 10 Excellent (+++), 0 Good (++), and 0 Poor (+)

Both modalities excelled in this final step.

**Figure 12:**
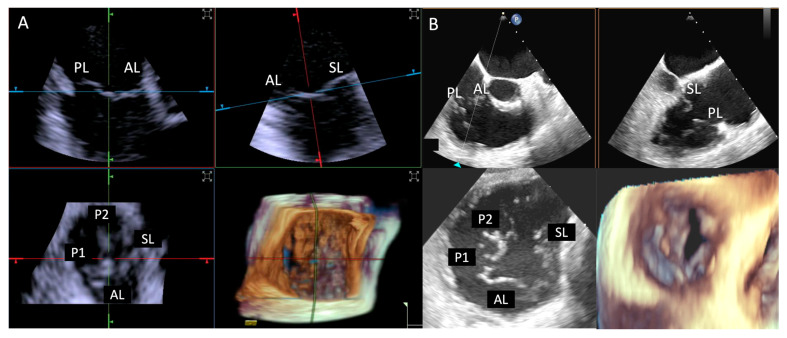
**Step 1: insights from Case 7**. The figure illustrates the comparative analysis of tricuspid valve evaluation during Step 1. In this case, both imaging modalities were rated as «excellent». TEE (**B**) images provide superior image quality with respect to 4D-ICE (**A**), showcasing TEE’s superior spatial and temporal resolution. Abbreviations: 4D-ICE: four-dimensional intracardiac echocardiography. AL: anterior leaflet. PL: posterior leaflet. SL: septal leaflet. TEE: transesophageal echocardiography.

When both imaging modalities were evaluated as equally effective in a procedural step, the quality of TEE images consistently outperformed that of 4D-ICE in every case. This finding underscores the superior spatial and temporal resolution still offered by TEE with respect to 4D-ICE (Figure 12).

Details of the step-by-step evaluation are presented in Table 4.

The performance of imaging modalities in cases requiring more than one edge-to-edge device was re-evaluated, focusing on Step 6 (grasping) and Step 7 (leaflet insertion), revealing similar challenges as in previous steps, such as shadowing from anterior structures affecting TEE. ICE appeared less impacted by the presence of the previously placed clip, maintaining clearer visualization.

**Figure 13:**
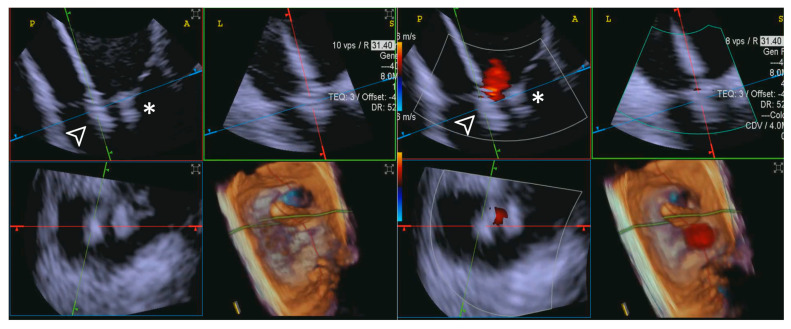
**Second edge-to-edge device implant**. The figure illustrates the capability of 4D-ICE to maintain optimal image quality while effectively visualizing the procedure, even during the implantation of a second edge-to-edge device (white arrowhead). In some cases, TEE can suffer from shadowing artifacts from the first device (*), whereas 4D-ICE does not usually suffer from significant shadowing artifacts, often ensuring continuous and clear visualization of the leaflets, device positioning, and overall procedural steps. This enables precise assessment of device interaction with the tricuspid valve and enhances procedural guidance without compromising image integrity. Abbreviations: 4D-ICE: four-dimensional intracardiac echocardiography. TEE: transesophageal echocardiography.

This observation was consistent even in patients without shadowing from anterior structures, where both ICE and TEE initially received an “Excellent” grading during the placement of the first edge-to-edge device, further highlighting ICE’s ability to maintain image quality despite the presence of previously placed clips (Figure 13).

#### 3.3.2. Quantitative Measurements Comparison

As far as quantitative measures are concerned, there were no statistically significant differences between 4D-ICE and TEE measurements for the septal leaflet (*p* = 0.677), anterior leaflet (*p* = 0.154), and posterior leaflet length (*p* = 0.332).

No statistically significant difference was found for annular perimeter measurements (*p* = 0.656).

Unlike the other parameters, the anteroposterior diameter showed a statistically significant difference between 4D-ICE and TEE measurements (*p* = 0.021). For the septolateral diameter, no statistically significant differences were found (*p* = 0.953).

No statistically significant differences were found between ICE and TEE measurements for the coaptation gap (*p* = 0.081), 2D-VC (*p* = 0.053), and final gradient, the latter being identical between 4D-ICE and TEE for all cases.

Details of measurements are presented in Supplementary Table S1.

## 4. Discussion

This study provides a comprehensive comparison between 4D-ICE and TEE in guiding tricuspid transcatheter edge-to-edge repair.

The step-by-step evaluation demonstrates that while both imaging modalities are valuable tools, their performance varies significantly depending on the image quality at each step.

Four-dimensional ICE achieved more “Excellent” ratings overall, indicating convincing utility in specific procedural steps. ICE’s intraprocedural advantages, such as proximity to the target structures and lack of external interference, likely contributed to its performance.

Conversely, TEE encountered challenges in several steps, notably during the steering and valve approach, grasping, and leaflet insertion, as shadowing artifacts from anterior cardiac structures likely impacted its accuracy during these procedural phases. Our small cohort of patients was composed of “highly complex” cases; the majority of cases (6/10) had a history of previous valve intervention (mitral or aortic valve), which could have affected TEE performance due to significant shadowing artifacts.

Nevertheless, TEE demonstrated reliability in the assessment of tricuspid valve anatomy and identification of target lesions, with adequate imaging in all cases and “Excellent” ratings comparable to ICE for certain steps.

When both imaging modalities were evaluated as equally effective in a procedural step, the image quality of TEE images consistently outperformed that of 4D-ICE in every case. This finding underscores the superior spatial and temporal resolution still offered by TEE. The higher resolution of TEE facilitates enhanced visualization of fine anatomical details and dynamic cardiac structures, which is particularly advantageous for complex interventional procedures. Conversely, while 4D-ICE offers the benefit of reduced invasiveness and real-time imaging from within the heart, its image quality is inherently limited by current technology. These limitations highlight the ongoing need for advancements in intracardiac echocardiography systems to match the resolution of TEE.

Quantitative analysis revealed no statistically significant differences in several critical parameters, including measurements of the tricuspid leaflets (anterior, septal, and posterior), annular perimeter, septolateral diameter, coaptation gap, and final gradient. However, the anteroposterior diameter showed a statistically significant difference between the two modalities (*p* = 0.021). This difference could stem from variations in imaging planes or resolution capabilities. Despite this, the lack of significant differences in other parameters reinforces the overall comparability of ICE and TEE for quantitative assessments.

These observations are consistent with prior studies [8,9], which highlighted the robustness of 4D-ICE in assessing leaflet morphology and device positioning during transcatheter interventions.

Davidson et al. [8] reported similar advantages of 4D-ICE in achieving precise anatomical assessments and procedural guidance during transcatheter tricuspid valve repair, emphasizing its potential to overcome the limitations of TEE in challenging cases [10,11].

It is important to acknowledge a significant limitation in the image quality comparison: Although echocardiographers were blinded to procedural outcomes, they were not blinded to the imaging technique (TEE vs. ICE), which was easily identifiable due to distinctive image characteristics. This introduces a potential bias in the qualitative assessment of image quality. Moreover, the subjective categorization of image quality (excellent, good, and poor) inherently limits the objectivity and reproducibility of this evaluation. These factors should be considered when interpreting comparative results.

While this study provides valuable insights, its small sample size (*n* = 10) limits the generalizability of the findings. As certain procedural steps benefited from improved visualization with 4D-ICE, our findings do not support definitive claims of superiority. Additionally, due to the low statistical power, this study does not allow for a valid demonstration of non-inferiority. Based on our findings, 4D-ICE appears to be a promising and complementary tool, particularly in selected patient populations or anatomies with sub-optimal TEE imaging.

Additionally, as a single-center study, the results may be influenced by operator expertise and institutional protocols. Future multicenter studies with larger cohorts are necessary to validate these findings and establish standardized protocols for integrating 4D-ICE into routine practice.

Future research should also focus on evaluating the long-term potential of 4D-ICE-only guided T-TEER, including durability of repair and residual tricuspid regurgitation. Moreover, the economic implications of incorporating 4D-ICE into routine practice should not be overlooked. Although ICE would avoid the use of conscious sedation, avoid general anesthesia, and shortening recovery, the economic benefit is partially offset by the high cost of single-use 4D-ICE catheters. Future cost-effectiveness analyses are needed in order to determine the economic feasibility of widespread 4D-ICE adoption.

## 5. Conclusions

This study highlights the strengths of 4D-ICE as a promising and reliable imaging modality for guiding T-TEER procedures. Its ability to provide detailed and unobstructed views during critical steps, coupled with its equivalence in quantitative measures, underscores its value as a viable complement or alternative to TEE in selected patient populations. By addressing the limitations in patients with sub-optimal TEE windows, 4D-ICE represents a significant advancement in the field of structural heart interventions. Future research and technological innovation will further define its role in clinical practice.

## Figures and Tables

**Table 1:** Baseline patient characteristics.

Variable	*N* = 10
Age (years)	77 ± 9.9
Female *n.* (%)	5 (50%)
BMI (kg/m^2^)	25 ± 5.58
Diabetes Mellitus, *n.* (%)	2 (20%)
Hypertension, *n.* (%)	9 (90%)
Hypercholesterolemia, *n.* (%)	4/40%)
Previous AMI *n.* (%)	0 (0%)
Previous PCI, *n.* (%)	1 (10%)
Previous CABG, *n.* (%)	0 (0%)
Previous valve intervention, *n.* (%) MV replacement, *n.* (%) M-TEER, *n.* (%) Other, *n.* (%)	6 (60%) 3 (30%) 2 (20%) 1 (10%)
Previous Stroke/TIA, *n.* (%)	0 (0%)
COPD, *n.* (%)	1 (10%)
History of AF/AFL, *n.* (%)	10 (100%)
Hb (g/dL)	13.5 ± 1.9
BNP (pg/mL)	246 ± 131
NYHA class III-IV, *n.* (%)	7 (70%)
Triscore (%)	19 ± 17
Triscore pure number	4.9 ± 1.96
ICD/CRT-D/P, *n.* (%)	1 (10%)
Loop Diuretics, *n.* (%)	10 (100%)
Beta-blockers, *n.* (%)	8 (80%)
ACE-I/ARBs, *n.* (%)	5 (50%)
ARNI, *n.* (%)	0 (0%)
MRA, *n.* (%)	6 (60%)
Digoxin, *n.* (%)	3 (30%)
SGLT-2-i, *n.* (%)	1 (10%)
NOAC, *n.* (%)	10 (10%)

Data are presented as means ± standard deviation (SD) for continuous variables and as counts (*n*) with percentages (%) for categorical variables. Abbreviations: ACE-I: Angiotensin-Converting Enzyme Inhibitor. AF: Atrial Fibrillation. AFL: Atrial Flutter. AMI: Acute Myocardial Infarction. ARB: Angiotensin II Receptor Blocker. ARNI: Angiotensin Receptor–Neprilysin Inhibitor. BMI: Body Mass Index. BNP: B-type Natriuretic Peptide. CABG: Coronary Artery Bypass Grafting. COPD: Chronic Obstructive Pulmonary Disease. CRT-D: Cardiac Resynchronization Therapy Defibrillator. CRT-P: Cardiac Resynchronization Therapy Pacemaker. Hb: Hemoglobin. ICD: Implantable Cardioverter Defibrillator. M-TEER: mitral transcatheter edge-to-edge repair. MRA: Mineralocorticoid Receptor Antagonist. MV: mitral valve. NOAC: Non-Vitamin K Antagonist Oral Anticoagulant. NYHA: New York Heart Association. PCI: Percutaneous Coronary Intervention. SGLT-2-i: Sodium-Glucose Co-Transporter-2 Inhibitor. TIA: Transient Ischemic Attack.

**Table 2:** Baseline echocardiographic parameters.

Variable	*N* = 10
TR severity:	
Severe TR, *n.* (%) Massive TR, *n.* (%) Torrential TR, *n.* (%)	9 (90%) 1 (10%) 0 (0%)
Mechanism of TR:	
Primary, *n.* (%) Secondary, *n.* (%) CIED-Related, *n.* (%)	2 (20%) 8 (80%) 0 (0%)
Atrial Functional TR, *n.* (%)	8 (80%)
RV EDAi (cm^2^/m^2^)	12.5 ± 4.2
RV FAC (%)	43.7 ± 5.5
TAPSE (mm)	18.3 ± 4.5
RVEDV (mL)	146 ± 52
RVEF (%)	45.8 ± 6.2
RV strain	21.5 ± 4.7
RA Area (cm^2^)	36.1 ± 15.8
sPAP (mmHg)	39.5 ± 7.2
TAPSE/PAPs (mm/mmHg) >0.36, *n.* (%) <= 0.36, *n.* (%)	0.47 ± 0.10 9 (90%) 1 (10%)
IVC size (mm)	21 ± 3
LA Volume index (mL/m^2^)	73.1 ± 62.4
LVEDV (mL)	90.5 ± 37.9
LVEDVi (mL/mq)	50.1 ± 20.3
LVEF (%)	56.3 ± 6.9
More than moderate MR, *n.* (%)	0 (0%)

Data are presented as means ± standard deviation (SD) for continuous variables, and as counts (*n*) with percentages (%) for categorical variables. Abbreviations: CIED: Cardiac Implantable Electronic Device. IVC: Inferior Vena Cava. LA: Left Atrium. LVEDV: Left Ventricular End-Diastolic Volume. LVEDVi: Left Ventricular End-Diastolic Volume index. LVEF: Left Ventricular Ejection Fraction. MR: Mitral Regurgitation. PAPs: Pulmonary Artery Pressures systolic. RA: right atrium. RV: Right Ventricle. RVEDV: Right Ventricular End-Diastolic Volume. RVEF: Right Ventricular Ejection Fraction. RV EDAi: Right Ventricular End-Diastolic Area index. RV FAC: Right Ventricular Fractional Area Change. TAPSE: Tricuspid Annular Plane Systolic Excursion. TR: tricuspid regurgitation. sPAP: Systolic Pulmonary Artery Pressure.

**Table 3:** Procedure-related.

Variable	*N* = 10
Main access—right femoral, *n.* (%)	10 (100%)
Closure system—suture-based VCDs, *n.* (%)	10 (100%)
Platform	
PASCAL, *n.* (%) TriClip, *n.* (%)	5 (50%) 5 (50%)
Number of necessary clips	
1, *n.* (%) 2, *n.* (%) 3, *n.* (%)	2 (20%) 7 (70%) 1 (10%)
Mean procedural time (min)	91.8 ± 13.9
Final regurgitation:	
mild, *n.* (%) mild–moderate, *n.* (%) moderate or more, *n.* (%)	8 (80%) 2 (20%) 0 (0%)
Complications during or after procedure, *n.* (%)	0 (0%)
Vascular complications, *n.* (%)	0 (0%)
Clip early detachment, *n.* (%)	0 (0%)

Data are presented as means ± standard deviation (SD) for continuous variables, and as counts (*n*) with percentages (%) for categorical variables. Abbreviations: VCDs: Vascular Closure Devices.

**Table 4:** Case by case feasibility comparison.

Case	Imaging	Step 1	Step 2	Step 3	Step 4	Step 5	Step 6	Step 7	Step 8
		*TV Valve Assessment*	*Target Lesion Identification*	*Steering and Approaching*	*Perpendicularity and Trajectory*	*Clocking*	*Grasping*	*Leaflets Insertion*	*Residual Jets Evaluation*
**1**	ICE	+++	+++	+++	+++	+++	+++	+++	+++
	TEE	+++	+++	+++	+++	+++	++	++	+++
**2**	ICE	++	+++	+++	+++	+++	+++	+++	+++
	TEE	+++	+++	+++	+++	+++	+++	+++	+++
**3**	ICE	+++	+++	+++	+++	+++	+++	+++	+++
	TEE	+++	+++	+	+	+++	+	+	+++
**4**	ICE	+++	+++	+++	+++	+++	+++	+++	+++
	TEE	++	+++	+	+	+++	++	+	+++
**5**	ICE	+++	+++	+++	+++	+++	+++	+++	+++
	TEE	++	+++	+++	+++	+++	++	++	+++
**6**	ICE	+++	+++	+++	+++	+++	+++	+++	+++
	TEE	++	+++	+	+	+++	+	+	+++
**7**	ICE	+++	+++	+++	+++	+++	+++	+++	+++
	TEE	+++	+++	+++	+++	+++	+++	+++	+++
**8**	ICE	+++	+++	+++	+++	+++	+++	+++	+++
	TEE	++	+++	+++	+++	+	+	+	+++
**9**	ICE	++	+++	+++	+++	+++	+++	+++	+++
	TEE	+++	+++	+++	+++	+++	+++	++	+++
**10**	ICE	+++	+++	+++	+++	+++	++	++	+++
	TEE	+++	+++	+++	+++	+++	+++	+++	+++

Excellent, green (+++): The image quality was deemed sufficient to complete the procedural step independently without the need for additional images from another modality. Good, orange (++): The image quality was adequate to complete the procedural step; however, verification with an alternative imaging modality could potentially enhance the outcome. Poor, red (+): The image quality was insufficient for completing the procedural step, necessitating the use of another imaging modality. The identified steps were as follows: Step number 1: assessment of tricuspid valve anatomy; Step number 2: identification of the target lesion; Step number 3: steering and valve approach; Step number 4: ensuring perpendicularity and correct trajectory; Step number 5: clocking; Step number 6: grasping; Step number 7: leaflet insertion; Step number 8: evaluation of residual regurgitant jets. Abbreviations: ICE: intracardiac echocardiography. TEE: transesophageal echocardiography. TV: tricuspid valve.

## Data Availability

The data presented in this study are available on request from the corresponding author.

## Supplementary Materials

The following supporting information can be downloaded at: https://www.mdpi.com/article/10.3390/jcdd12050165/s1, Video S1: Step 7: insights from Case 6. The video illustrates the comparative analysis of leaflet insertion evaluation during Step 7. In this case, the superior performance of 4D-ICE (A) is shown, as compared to TEE (B), due to shadowing artifacts derived from anterior structures. Abbreviations: 4D-ICE: four-dimensional intracardiac echocardiography. TEE: transesophageal echocardiography. Table S1. Comparison of quantitative measurements obtained using ICE and TEE during T-TEER in 10 cases. Each row represents a specific parameter measured: septal leaflet length, anterior leaflet length, posterior leaflet length, annular perimeter, AP diameter, SL diameter, coaptation gap, 2D-VC basal width, and final pressure gradient. Measurements are displayed for both ICE and TEE imaging modalities across all cases. *p* values indicate the statistical comparison between the two modalities for each parameter. “N.A.” denotes measurements not available for certain cases. Abbreviations: AP: anteroposterior. ICE: intracardiac echocardiography. SL: septolateral. TEE: transesophageal echocardiography. VC: vena contracta.
