# Supplementary material for: Early Experience with Acuson AcuNav 4D-ICE to Guide Transcatheter Tricuspid Edge-to-Edge Repair: 4D Intracardiac Echocardiography Compared to Transesophageal Echocardiography
Source: J Cardiovasc Dev Dis. 2025 Apr 23;12(5):165. doi: 10.3390/jcdd12050165 (PMC12112266; doi:10.3390/jcdd12050165)
Supplement: Supplementary file 1 [file jcdd-12-00165-s001.zip › Supplementary Table S1.pdf]

Supplementary Table S1

Comparison of measurements obtained using ICE and TEE during T-TEER in 10 cases. Each row represents a specific parameter measured: septal leaflet length, anterior leaflet length, posterior leaflet length, annular perimeter, AP diameter, SL diameter, coaptation gap, 2D VC basal width, and final pressure gradient. Measurements are displayed for both ICE and TEE imaging modalities across all cases. P values indicate the statistical comparison between the two modalities for each parameter. "N.A." denotes measurements not available for certain cases.

AP: Anteroposterior. ICE: Intracardiac Echocardiography. SL: Septolateral. TEE: Transesophageal Echocardiography. VC: Vena Contracta.

Case	1		2		3		4		5		6		7		8		9		10		
**Imaging modality**	**ICE**	**TEE**	**ICE**	**TEE**	**ICE**	**TEE**	**ICE**	**TEE**	**ICE**	**TEE**	**ICE**	**TEE**	**ICE**	**TEE**	**ICE**	**TEE**	**ICE**	**TEE**	**ICE**	**TEE**	*P Value*
**Septal leaflet (mm)**	13.6	13.6	20.0	21.0	18.0	16.0	18.3	17.9	18.0	12.0	18.0	12.0	15.0	18.0	18.0	22.0	17.0	16.0	14.0	15.0	**p=0.667**
**Anterior leaflet (mm)**	17.5	20.6	26.7	28.0	23.5	21.5	27.0	22.5	28.0	23.7	30.0	28.0	22.0	21.0	35.0	29.0	27.0	28.0	22.0	22.0	**p=0.154**
**Posterior Leaflet (mm)**	24.7	24.0	26.1	27.8	25.0	19.0	24.0	21.8	26.0	21.2	21.0	20.0	20.0	25.0	32.0	27.0	30.0	29.0	17.0	18.0	**p=0.332**
**Perimeter (mm)**	147	149	155	164	142	137	125	129	137	150	145	N.A.	130	135	159	N.A.	150	162	127	130	**p=0.656**
**AP diameter (mm)**	55.3	52.7	51.5	51.9	30.0	36.0	28.0	36.0	38.0	46.0	40.0	50.0	34.0	45.0	49.0	51.0	40.0	50.0	46.0	46.0	**p=0.021**
**SL diameter (mm)**	42.0	45.0	44.0	46.3	50.0	48.0	48.0	45.0	47.0	48.0	50.0	50.0	47.0	40.0	52.0	51.0	51.0	54.0	34.0	32.0	**p=0.953**
**Coaptation gap (mm)**	4.0	4.0	4.0	5.0	3.0	3.0	7.0	7.0	2.0	2.0	6.0	7.0	5.0	5.0	5.0	5.0	10.0	11.0	6.0	7.0	**p=0.081**
**2D VC basal (mm)**	8.0	8.0	6.0	8.0	6.0	7.0	10.0	10.0	6.0	7.0	9.0	8.0	7.0	8.0	8.0	10.0	8.0	10.0	7.0	8.0	**p=0.053**
**Final gradient (mmHg)**	1.0	1.0	1.0	1.0	1.0	1.0	1.0	1.0	1.0	1.0	1.0	1.0	1.0	1.0	1.0	1.0	2.0	2.0	2.0	2.0	N.A
